# Development and validation of a scoring system for predicting cancer patients at risk of extended-spectrum b-lactamase-producing *Enterobacteriaceae* infections

**DOI:** 10.1186/s12879-020-05280-4

**Published:** 2020-07-31

**Authors:** Alvaro J. Martínez-Valencia, Brian J. Gómez Martínez, Anita M. Montañez Ayala, Katherin García, Ricardo Sánchez Pedraza, Leydy P. Jiménez Cetina, Julio C. Gómez Rincón, Sonia I. Cuervo Maldonado

**Affiliations:** 1 Departamento de Medicina Interna. Bogotá, Universidad Nacional de Colombia, Bogotá, D.C., Colombia; 2 Grupo de Enfermedades Infecciosas, Instituto Nacional de Cancerología E.S.E, Bogotá, D.C., Colombia; 3 Laboratorio de Microbiología, Instituto Nacional de Cancerología E.S.E, Bogotá, D.C., Colombia; 4 Grupo en Enfermedades Infecciosas en Cáncer y Alteraciones Hematológicas (GREICAH), Bogotá, D.C., Colombia

**Keywords:** Beta-lactamases, Extended-spectrum β-lactamase producing *Enterobacteriaceae*, Cancer, Prediction, Risk stratification

## Abstract

**Background:**

Extended-spectrum beta-lactamase producing *Enterobacteriaceae* (ESBL-PE) infections are frequent and highly impact cancer patients. We developed and validated a scoring system to identify cancer patients harboring ESBL-PE at the National Institute of Cancer of Colombia.

**Methods:**

We retrospectively analyzed medical records of 1695 cancer patients. Derivation phase included 710 patients admitted between 2013 to 2015, ESBL-PE positive culture (*n* = 265) paired by month and hospitalization ward with Non-ESBL-PE (*n* = 445). A crude and weighted score was developed by conditional logistic regression. The model was evaluated in a Validation cohort (*n* = 985) with the same eligibility criteria between 2016 to 2017.

**Results:**

The score was based on eight variables (reported with Odds Ratio and 95% confidence interval): Hospitalization ≥7 days (5.39 [2.46–11.80]), Hospitalization during the previous year (4, 87 [2.99–7.93]), immunosuppressive therapy during the previous 3 months (2.97 [1.44–6.08]), Neutropenia (1.90 [1.12–3.24]), Exposure to Betalactams during previous month (1.61 [1.06–2.42]), Invasive devices (1.51 [1.012–2.25]), Neoplasia in remission (2.78 [1.25–1.17]), No chemotherapy during the previous 3 months (1.90 [1.22–2.97]). The model demonstrated an acceptable discriminatory capacity in the Derivation phase, but poor in the Validation phase (Recipient Operating Characteristic Curve: 0.68 and 0.55 respectively).

**Conclusions:**

Cancer patients have a high prevalence of risk factors for ESBL-PE infection. The scoring system did not adequately discriminate patients with ESBL-PE. In a high-risk population, other strategies should be sought to identify patients at risk of resistant ESBL-PE infection.

## Summary

Cancer patients have a high prevalence of risk factors for ESBL-PE infection. The developed scoring system did not adequately discriminate patients with ESBL-PE. In high-risk population other strategies should be sought to identify patients at risk of ESBL-PE infection.

## Introduction

Infections caused by ESBL-PE (*Escherichia coli, Klebsiella spp*. and *Proteus mirabilis*) are a major clinical problem and represent a growing proportion of infections acquired in the community and associated with health care worldwide [1–4].

The delay in the initiation of adequate antibiotic treatment against infections caused by ESBL-PE is associated with increased morbidity, duration of hospitalizations, mortality and treatment-related costs [5].

Carbapenems are considered the antibiotics of choice for the treatment of ESBL-PE infections given their stability against the hydrolytic activity of ESBL. However, excessive use of carbapenems promotes the selection of microorganisms resistant to these antibiotics, leaving few therapeutic options to treat infections by resistant microorganisms [6–8].

Identification of patients at risk of ESBL-PE infections could allow timely and adequate selection of appropriate empirical antibiotic treatment, reducing treatment failure, complications, antibiotic costs and inappropriate use of carbapenems with the risk of selecting resistant microorganisms [9, 10].

Cancer patients represent an intrinsically vulnerable population to infections, particularly to ESBL-PE. It is recognized that the depressed immune system and frequent lesions in the gastrointestinal mucosa and skin barriers due to surgical interventions, invasive devices or cytotoxic chemotherapy, facilitate the translocation or invasion of tissues and bloodstream by ESBL-PE [11–14]. In addition, frequent use of antibiotics as prophylaxis or treatment has also been linked to increased ESBL-PE infections in cancer patients [15–17].

Several clinical scoring tools have been developed to identify patients at risk of ESBL-PE infection, with different populations and heterogeneous findings [18], although none of them evaluate specifically patients with solid or hematologic malignancies.

The objective of this study was to develop and validate a reliable and easy-to-use clinical scoring system to identify patients with solid or hematologic malignancies with a high risk of ESBL-PE infections at the National Cancer Institute of Colombia (Instituto Nacional de Cancerología).

## Methods

### Study design and study site

A retrospective, analytical study was conducted in cancer patients with documented microbiological isolation during hospitalization at the National Cancer Institute of Colombia; an institution located in Bogotá with 180 beds and an admission rate of 14,000 patients/year, national reference center for solid and hematological cancer care in the country.

A case-control study (Derivation phase) was carried out to identified risk factors in cancer patients for microbiological isolation of ESBL-PE in any clinical sample, then a scoring system was developed assigning weight to each factor. The scoring system was evaluated in a cohort admitted on a different date (Validation phase).

### Inclusion criteria and case definition

Patients admitted to the hospital of any sex and age, with a confirmed oncological diagnosis (independent of stage or activity of cancer) and microbiological isolation of *Enterobacteriaceae* in any clinical sample since admission to the hospital were included. Patients with prior isolation of ESBL-PE or incomplete information of the study variables were excluded. Only the first culture (index) was taken into account in case of more than one isolation. The cases consisted of patients admitted between 1 January 2013 and 31 December 2015, with ESBL-PE isolation; two controls were sought for each case with isolation of non-ESBL-PE, paired for the same month and hospitalization ward at the time of index culture. Patients included in the Validation phase were admitted between 1 January 2016 and 31 December 2017, with the same eligibility criteria. Patients were only included in a single phase of the study.

### Collection of data and variables

Patients were selected from the microbiology laboratory database and the inclusion performed chronologically until the sample size was completed. The closest control to the date of the index culture of the corresponding case was selected. Information was obtained from laboratory reports and electronic medical records. The study variables included demographic information (sex, admission site, hospitalization room), cancer-related (hematological or solid malignancy, active or remission malignancy according to the medical record of the treating physician), comorbidities (liver disease, kidney disease, diabetes mellitus, lung disease chronic obstructive, heart failure, cerebrovascular disease, dementia, connective tissue disease, acquired immunodeficiency syndrome), Charlson comorbidity index [19], healthcare-related (hospitalization during the last year, length of hospital stay (≥7 days), immunosuppression [prednisone 7.5 mg per day, tacrolimus, sirolimus, cyclosporine, mycophenolate, during the previous 2 weeks] radiation therapy in the previous 3 months, chemotherapy in the previous 3 months, neutropenia at the time of culture, use of invasive devices [central venous catheter, bladder catheter, surgical drains or nasogastric tube], surgeries during the last year, antimicrobial use in the last month) and microbiological (isolated microorganism, antimicrobial susceptibility). To reduce errors in data capture, the information was recorded electronically in REDCap® and was independently verified by an institutional monitor to identify discrepancies.

### Microbiological analysis

Cultures were ordered according to criteria of the attending physician and carried out following institutional protocols. The Vitek 2 system (bioMerieux, Inc., Hazelwood, MO) or Phoenix (Becton Dickinson Microbiology Systems) fluorimetry and colorimetry for species identification and colorimetry and turbidimetry for susceptibility assessment was used. Phenotypic detection was performed according to recommendations and cut-off points for cefotaxime and ceftazidime and the combination of both with clavulanic acid according to the Vitek 2 and Phoenix 100 panel following the recommendations and current cut-off points of CLSI for Colombia without confirmation by molecular biology [20].

### Sample size

For the derivation phase, a number of 10 events per variable included in the model was taken into account [21]. A total of 28 study variables were evaluated, 280 cases (ESBL-PE event) and 560 controls were estimated to be included, with a ratio of cases to controls of 1:2. For the validation phase, 985 patients were included, taking into account an estimated sensitivity and specificity of the scoring system of 80%, an approximate prevalence of ESBL-PE infections of 25% [3, 22] and an accuracy of 5% around the estimator.

### Statistical analysis

For categorical variables absolute and relative frequencies were calculated, the Chi2 test or Fisher’s exact test was applied to estimate differences between groups. For continuous variables, normality assumptions were validated from the Shapiro-Wilk test. In variables with normal distribution, averages and standard deviations were calculated, otherwise, medians and ranges were estimated. To establish comparisons between groups, the T-Student or U-Mann-Whitney test was applied. In the bivariate analysis, those variables with a significance value of less than 0.15 were incorporated into the multivariable conditional logistic regression analysis. Variables incorporated in the final model were selected using a “stepwise” strategy, maintaining probability input values of 0.15. The final model was transformed into a raw score: assigning an equal score for each variable, and also a weighted score: each variable with a score calculated by dividing each regression coefficient by half of the smallest coefficient and rounded to the nearest integer [23]. The discriminatory power of the model was determined by calculating sensitivity, specificity and Area Under the Receiver Operating Characteristic Curve (AUC-ROC). The optimal cutting points were established by the method proposed by Liu [24]. Additionally, sensitivity, specificity, positive and negative predictive values (PPV and NPV, respectively) were estimated with different cut-off points. Except for the “stepwise” procedures, 5% significance values were used and two-tailed hypotheses were tested. All statistical analyses were performed with Stata 13®.

### Ethical considerations

The study was approved by the Institutional Review Boards of the Universidad Nacional de Colombia and Instituto Nacional de Cancerología. Written informed consent was not required.

## Results

### Derivation phase

A total of 265 cases with a positive culture for ESBL-PE met the eligibility criteria. For 180 cases their respective 2 paired controls were found, for the remaining 85 cases, only 1 paired control met all the eligibility criteria. In total, 710 patients were included in the derivation phase. The median age for cases was 56 years, 56% were men, 231 (87%) were admitted through the emergency department, the majority had a solid tumor (70%) and active malignancy (92%). *E. coli and Klebsiella spp*. represented 98% of the microbiological isolates of the cases (Table 1).

**Table 1:** Characteristics of cancer patients with *Enterobacteriaceae* isolation. Derivation Phase

**Total, No. (%)**	**Cases** **(ESBL-PE)*n* = 265 (37)**	**Controls** **(non-ESBL-PE)*n* = 445 (63)**	***p***
**Clinical characteristics**			
Age, median (IQR), years	56 (39–67)	60 (47–70)	0.009
Age ≥ 70 years	49 (18)	114 (25)	0,029
Men	148 (56)	286 (64)	0.026
Admission as an outpatient	231 (87)	425 (96)	0.000
Solid tumor	186 (70)	336 (76)	0.120
Hematological malignancy	79 (30)	109 (34)	
Active neoplasia	245 (92)	428 (96)	0.031
Neoplasia in remission	20 (8)	17 (4)	
**Comorbidities**			
Acute myocardial infarction	6 (1,5)	7 (2,2)	0,050
Symptomatic heart failure	5 (1,8)	9 (2)	0,900
Peripheral arterial disease	1 (0,38)	1 (0,22)	0,711
Cerebrovascular disease	2 (0,7))	3 (0,6))	0,901
Dementia	2 (0,7)	4 (0,9)	0,839
Chronic obstructive pulmonary disease	14 (5,2)	18 (4,0)	0,442
Connective tissue disease	2 (0,7)	8 (1,8)	0,254
Peptic acid disease	2 (0,7)	3 (0,6)	0,901
Chronic liver disease	5 (1,8)	6 (1,3)	0,574
Mellitus diabetes	18 (6,7)	44 (9,8)	0,158
Hemiplegia	9 (3,4)	12 (2,7)	0,595
Renal disease	20 (7,5)	28 (6,2)	0,519
Metastasis	85 (32)	135 (30)	0,628
AIDS	4 (1,5)	3 (0,6)	0,276
Charlson comorbidity index ≥4	104 (39)	167 (36)	0,649
**Medical interventions**			
Hospitalization during previous year	223 (84)	264 (59)	0.000
Prolonged hospitalization (≥7 days)	119 (45)	141 (32)	0.000
Immunosuppressive therapy^a^	27 (10)	19 (4)	0,002
Radiation therapy^a^	23 (9)	43 (10)	0.662
Chemotherapy^a^	89 (34)	183 (41)	0.046
Neutropenia at the time of culture	59 (22)	68 (15)	0.019
Surgical procedures during previous year	130 (49)	168 (38)	0.003
Invasive devices at the time of culture^b^	146 (55)	177 (40)	0.000
Urinary catheter during last month	93 (35)	127 (29)	0,068
**Antibiotic use during previous month to index culture**			
Any antibiotic	102 (38)	108 (24)	0,000
Beta-lactams	80 (31)	82 (18)	0,000
Aminoglycosides	5 (2)	2 (0,4)	0,108
Quinolones	10 (3,8)	8 (1,8)	0,136
Carbapenemic	26 (10)	16 (3,6)	0,001
Cotrimoxazole	11 (4)	7 (1,5)	0,045
Others	21 (8)	23 (5)	0,125
**Isolate source**			
Blood	63 (23)	86 (19)	0.384
Urinary tract	142 (54)	272 (61)	
Surgical wound	47 (18)	68 (15)	
Skin and soft tissues	3 (1)	4 (1)	
Lower respiratory tract	10 (4)	15 (3)	
**Isolated microorganism**			
*E. coli.*	166 (63)	299 (67)	0.000
*Klebsiella spp.*	92 (35)	80 (18)	
*Proteus spp.*	3 (1)	55 (12)	
Others	4 (2)	11 (2)	

^a^ during the previous 3 months, ^b^central venous catheter, dialysis catheter, surgical drains, bladder catheter, nephrostomy, nasogastric tube

The multivariable conditional logistic regression analysis showed eight variables independently associated with the microbiological isolation of ESBL-PE: Hospitalization during the last year (*p* = 0.000), Prolonged hospitalization ≥7 days (p = 0.000), Immunosuppressive therapy during the 3 months previous (*p* = 0.003), Neutropenia (*p* = 0.017), Exposure to Beta-lactam drugs in the previous month (*p* = 0.022), Use of invasive devices (*p* = 0.038), Neoplasm in remission (*p* = 0.012) and No use of chemotherapy in the last 3 months (*p* = 0.004). A crude and weighted scoring system was constructed with the identified variables (Table 2).

**Table 2:** Risk factors for ESBL-PE isolation. Derivation Phase

**Variable**	**Adjusted Odds ratio (IC 95%)**	***p***	**Regression coefficient**	**Weighted score**	**Crude score**
Prolonged hospitalization (≥7 days)	5,39 (2,46 – 11,80)	0.000	1,68	8	1
Hospitalization during previous year	4,87 (2,99 – 7,93)	0.000	1,58	7	1
Immunosuppressive therapy^a^	2,97 (1,44 – 6,08)	0,003	1,08	5	1
Neutropenia	1,90 (1,12 – 3,24)	0,017	0,64	3	1
Beta-lactams during the previous month	1,61 (1,06 – 2,42)	0,022	0,47	2	1
Invasive devices at the time of culture^b^	1,51 (1,01 – 2,25)	0,038	0,41	2	1
Neoplasia in remission	2,78 (1,25 – 6,17)	0,012	1,02	5	1
No chemotherapy^a^	1,90 (1,22 – 2,97)	0,004	0,64	3	1

^a^ during the previous 3 months, ^b^central venous catheter, dialysis catheter, surgical drains, bladder catheter, nephrostomy, nasogastric tube

The crude score achieved an acceptable ability to discriminate patients with ESBL-PE isolation from patients without ESBL-PE (AUC-ROC: 0.684 95% CI 0.646–0.722). The weighted score achieved a similar capacity (AUC-ROC: 0.687 95% CI 0.648–0.726). For simplicity, it was decided to apply the crude score in the validation cohort.

### Validation phase

Consecutively, from 1 January 2016 to 26 December 2017, all patients were included until the sample size of 985 cancer patients with a positive culture for Enterobacteriaceae was completed. More than half of patients were older than 58 years, men represented 39%, the majority (51%) were admitted through the emergency department. *E. coli and Klebsiella spp.* accounted for 83% of microbiological isolates. The prevalence of ESBL-PE isolates was 19%. In the Validation cohort, there was a larger proportion of women (*p* = 004), lower frequency of hospitalization during the last year (*p* = 0.000), lower frequency of use of immunosuppressive therapy (*p* = 0.001) and lower frequency of isolation of *Klebsiella spp.* (*p* = 0.000) (Table 3).

**Table 3:** Comparison of patient in Derivation and Validation phase

**Total, No. (%)**	**Derivation**			**Validation**		
	**Cases** **(ESBL-PE)** ***n* = 265 (37)**	**Controls** **(non-ESBL-PE)** ***n* = 445 (63)**	***p***	**ESBL-PE** ***n*** **= 188 (19)**	**non-ESBL-PE** ***n*** **= 797 (81)**	***p***
Age, median (IQR), years	56 (39–67)	60 (47–70)	0.009	55 (38–67)	59 (45–70)	0.009
Men	148 (56)	286 (64)	0.026	80 (43)	309 (39)	0.340
Emergency room at index culture	231 (87)	425 (96)	0.000	93 (59)	405 (51)	0.850
Prolonged hospitalization (≥7 days)	119 (45)	141 (32)	0.000	72 (38)	244 (30)	0.042
Hospitalization during previous year	223 (84)	264 (59)	0,000	129 (69)	542 (68)	0.871
Immunosuppressive therapy^a^	27 (10)	19 (4)	0.002	4 (2)	10 (1)	0.363
Chemotherapy^a^	89 (34)	183 (41)	0.046	79 (42)	330 (41)	0.877
Invasive device at the time of culture^b^	146 (55)	177 (40)	0.000	98 (52)	348 (43)	0.036
Neoplasia in remission	20 (8)	17 (4)	0.031	11 (6)	30 (4)	0.197
Neutropenia	59 (22)	68 (15)	0.019	28 (15)	97 (12)	0.313
Beta-lactam use	80 (31)	82 (18)	0,000	49 (26)	99 (12)	0.000
**Isolate source**						
Blood	63 (23)	86 (19)	0.384	30 (16)	154 (19)	0.651
Urinary tract	142 (54)	272 (61)		123 (65)	485 (61)	
Surgical wound	47 (18)	68 (15)		11 (6)	62 (8)	
Skin and soft tissues	3 (1)	4 (1)		17 (9)	62 (8)	
Lower respiratory tract	10 (4)	15 (3)		7 (4)	34 (4)	
**Isolated microorganism**						
*E. coli.*	166 (63)	299 (67)	0.000	134 (71)	505 (63)	0.002
*Klebsiella spp.*	92 (35)	80 (18)		33 (18)	148 (19)	
*Proteus spp.*	3 (1)	55 (12)		5 (3)	94 (12)	
Others	4 (2)	11 (2)		16 (9)	50 (6)	

^a^ during the previous 3 months, ^b^central venous catheter, dialysis catheter, surgical drains, bladder catheter, nephrostomy, nasogastric tube

### Scoring system application

**Fig. 1:**
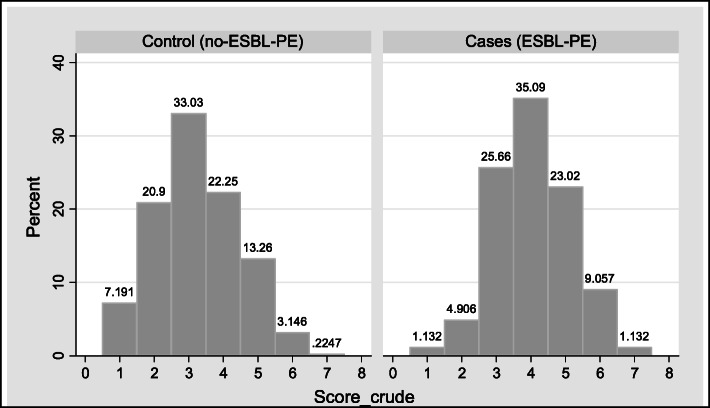
Distribution of crude score. Derivation Phase

In the Derivation phase, cases had an average and standard deviation (SD) of 4.05 +/− 1.11 points and controls 3.23 +/− 1.23 points (*p* = 0.000) (Fig. 1). The crude score with the best operating performance was ≥4 points, with a sensitivity of 68%, specificity of 61% and an accuracy of 63%.

**Fig. 2:**
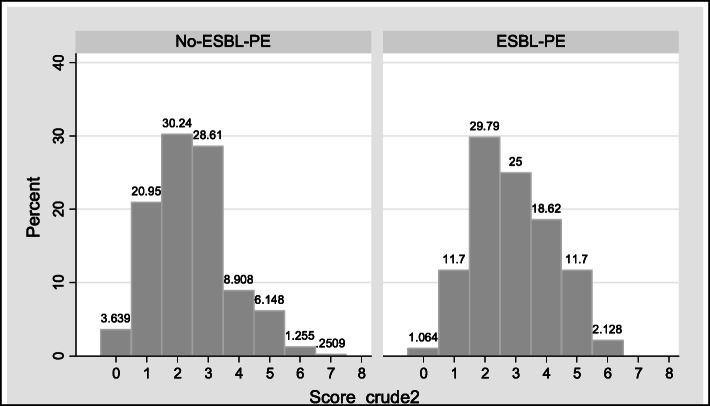
Distribution of crude score. Validation Phase

**Fig. 3:**
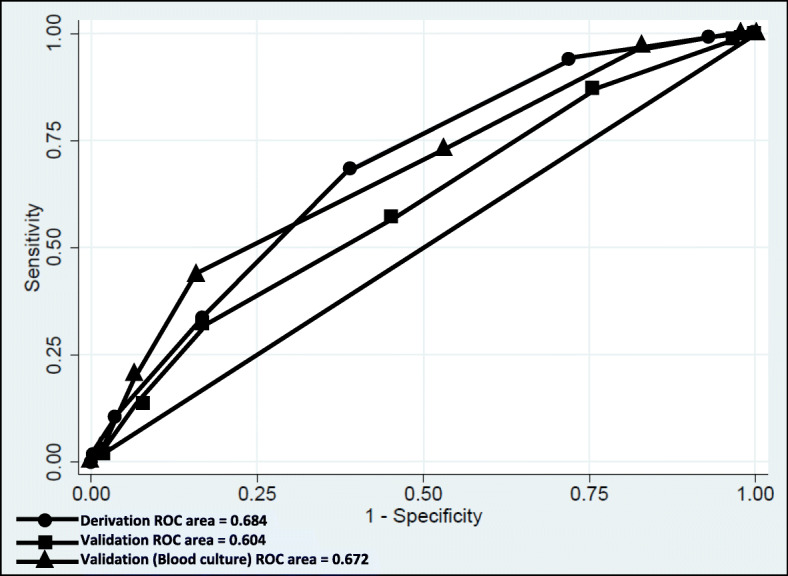
Receiver Operational Characteristic Curve Analysis with crude score. Derivation and validation phase

In the Validation phase, patients with ESBL-PE had an average of 2.9 +/− 1.3 points and patients with non-ESBL-PE 2.4 +/− 1.2 points (p = 0.000) (Fig. 2). Using the cut-off point ≥4 points, a sensitivity of 32%, a specificity of 83% and accuracy of 73% was obtained (Table 4). The crude score achieved an acceptable ability to discriminate patients with ESBL-PE isolation (AUC-ROC: 0.6042 95% CI 0.560–0.647) (Fig. 3).

**Table 4:** Classification of patients according to the crude score in the Derivation and Validation phase

**Crude score**	**Derivation** **Number of patients (%)**			**Validation** **Number of patients (%)**		
	**Cases**	**Controls**	**Total**	**ESBL-PE**	**Non ESBL-PE**	**Total**
0	0 (0)	0 (0)	0	2 (1)	29 (3)	31
1	3 (1)	32 (7)	35	22 (11)	167 (20)	189
2	13 (5)	93 (20)	106	56 (29)	241 (30)	297
3	68 (25)	147 (33)	215	47 (25)	228 (28)	275
4	93 (35)	99 (22)	192	35 (18)	71 (9)	106
5	61 (23)	59 (13)	120	22 (11)	49 (6)	71
6	24 (9)	14 (3)	38	4 (2)	10 (1)	14
7	3 (1)	1 (0)	4	0 (0)	2 (0)	2
Total	265	445	710	188	797	985

Subgroup analysis of blood cultures (*n* = 184) within the Validation phase showed an average of 3.36 +/− 1.27 points for ESBL-PE and 2.59 +/− 1.21 for non- ESBL-PE (*p* = 0.002). With the cut-off point ≥4 points, a sensitivity of 43%, specificity of 83% and accuracy of 77% were obtained. The crude score achieved an acceptable ability to discriminate patients with ESBL-PE isolation (AUC-ROC: 0.6722 95% CI 0.569–0.774) (Supplement).

## Discussion

ESBL-PE infections have increased in recent years and are a major cause of hospital and community-acquired infections [1, 3, 4, 12, 25, 26]. The carrier status in the healthy population is estimated at 14% worldwide, but in some regions, it can be as high as 46% [2]. Colonization status increases in populations at higher risk such as patients with solid or hematological malignancies, with a prevalence of 19% [25]. In Colombia, community-acquired urinary tract infections produced by *Enterobacteriaceae* with resistance to third-generation cephalosporins range from 3.4 to 17.2% [4].

Colonization by ESBL-PE is an important risk factor for subsequent infection or bacteremia by these microorganisms [12, 17, 27]. Other clinical characteristics of the patients, the epidemiological environment and the healthcare-related procedures are recognized as risk factors for ESBL-PE infection [9].

This study evaluated risk factors for isolation of ESBL-PE in cancer patients, derived and validated a scoring system to quickly identify these patients. The multivariable analysis identified eight variables associated with microbiological isolation of ESBL-PE, these included hospitalization in the last year, prolonged hospitalization greater than 7 days, immunosuppressive therapy by glucocorticoids or calcineurin inhibitors, presence of neutropenia, use of invasive devices, exposure to beta-lactam drugs in the previous month, remission neoplasia and no use of chemotherapy in the last 3 months. These factors confirm some already identified in previous studies [9]. The developed score includes simple variables to be evaluated clinically upon the first contact with the patient.

The crude scoring system achieved an acceptable ability to discriminate patients with ESBL-PE. Operational performance was better for the Derivation Phase; however, it fell in the Validation Phase (ROC-AUC 0.68 and 0.60). The cut-off point ≥4 points selected by the Liu method [24] that maximizes sensitivity and specificity showed generally low values in both phases (sensitivity 68 and 32%, specificity 61 and 83% respectively), also, the accuracy was low, with misclassification of 30%.

The scoring systems can not only be measured with the capacity of maximum discrimination with the ROC-AUC because the objective of their implementation must also be taken into account. If the goal is to screen hospitalized patients to determine their risk of ESBL-PE infection, a cut-off point with maximum sensitivity should be sought to capture the majority of patients at risk, with the price of lowering specificity; in this scenario, patients would receive treatment with carbapenems and contact isolation. The limitation of this approach is that many patients without ESBL-PE infection would end up receiving broad-spectrum antibiotics and contact isolation without needing. On the other hand, if the objective is to guide antibiotic therapy, specifically to avoid the indiscriminate use of carbapenems, a cut-off point with low sensitivity and high specificity should be sought [28, 29].

In Tumbarello study [23], the cut-off point of at least 3 points had high sensitivity (93%) and NPV (97%), but low specificity (62%) and PPV (45%); when the cut-off point rose to 6, sensitivity and NPV decreased to 63 and 88%, but specificity and PPV increased to 95 and 80%. In the Duke model [30], with a cut-off point of 8 points, good specificity and PPV (95 and 79%, respectively) were obtained, but low sensitivity (58%). In the Kengkla study [31] the 9-point cut-off obtained moderate sensitivity (74%) and NPV (68%) with inadequate specificity (66%). The most recent model proposed by Lee [32] obtained high sensitivity (84%) and specificity (92%) using a cut-off greater than 2 points. These studies included different populations and variables.

In the present study, setting the highest sensitivity (cut-off point ≥1) in the Derivation and Validation phases, showed that majority of patients had at least 1 risk factor (sensitivity of 100 and 99% respectively), but with a high percentage of false positives (100 and 97%). This makes the scale impractical to exclude patients without risk of ESBL-PE infection and would overestimate the empirical use of carbapenems and the need for contact isolation. Maximizing specificity (cut-off point ≥7) allows to identify patients without ESBL-PE (100% specificity), but only 1% of patients with ESBL-PE are captured, with low capacity to select patients at high risk of ESBL-PE. This way, a large number of patients who may require carbapenem treatment would not receive it and therefore the risk of mortality could increase. It is considered that with this scoring system there is no good cut-off point to predict a high risk of ESBL-PE infection nor to make decisions about the prescription of empirical antibiotics.

Previous described scoring systems may have better discriminatory performance because of the inclusion of more selected populations like the inclusion of patients within the first 48 h of admission, only with *E. coli* infection or presence of bacteremia [18]. This study included patients of all age ranges, patients with infection or colonization, all *Enterobacteriaceae* species, community and hospital-acquired infections, as well as infection of any organ. These characteristics reflect better the behavior of infections in the real life of an institution to make the results more generalizable. The subgroup analysis of blood cultures reflected similar performance to the whole validation cohort (Supplement).

The Validation cohort showed that the population attending the National Institute of Cancer of Colombia had a higher prevalence of ESBL-PE than the ambulatory population in Colombia, especially *E.coli* (19% vs. 6.4%) [4, 22]. Also, this population is highly morbid, approximately 70% required hospitalization in the last year, 40% received chemotherapy in the last 3 months, 45% had an invasive device and 25% was exposed to antibiotics in the last month.

In the context of patients with cancer and immunosuppression, the current therapeutic behavior is oriented to establishing the patient’s risk group and beginning empirical treatment with broad-spectrum antibiotics until microbiological isolation is achieved. This approach has been proposed recently, based on observational studies where patients are classified according to the immune status, source of infection and severity of disease presentation [7, 8]. For immunosuppressed patients (e.g. neutropenia, leukemia, lymphoma, HIV, solid organ or hematopoietic cell transplantation, cytotoxic chemotherapy or steroid use) and severely ill patients (high Pitt or APACHE II score, need for ICU or presence of septic shock) the beginning of empirical treatment with carbapenems has been recommended [8].

The results of this study allow us to conclude that in patients at high risk for ESBL-PE infections such as cancer patients, the risk score fails to adequately discriminate the patients and therefore other methods should be evaluated to early identify patients with ESBL-PE infections and to guide antibiotic therapy [33, 34].

The limitations of the study include its retrospective nature, historic medical records as source of information; the lack of inclusion of some specific variables of cancer patients (type of neoplasia, type of chemotherapy, ECOG and Karnofsky scores) which could have provided more information or allowed a better classification of patients; the absence of distinction between infection vs colonization, variables that are retrospectively difficult to discriminate and are proposed to be validated prospectively and non-discrimination between infection from the community or healthcare-associated. Finally, it is single specialized cancer institution in Colombia and therefore the results are not generalizable to other institutions with different characteristics. The findings of the neoplasia in remission and absence of chemotherapy in the previous 3 months as risk factors for ESBL-PE are inconsistent with previous studies and warrant confirmation in the context of cancer patients.

## Conclusions

Cancer patients have a high prevalence of risk factors for ESBL-PE infection. The scoring system did not adequately discriminate patients with ESBL-PE. In a high-risk population, other strategies should be sought to identify patients at risk of resistant ESBL-PE infection.

## Supplementary information

**Additional file 1.**

## Data Availability

The datasets used and/or analyzed during the current study is available from the corresponding author on reasonable request.

## Abbreviations

ESBL-PE: Extended-spectrum beta-lactamase producing Enterobacteriaceae
OR: Odds Ratio
AUC-ROC: Area Under the Receiver Operating Characteristic Curve
PPV: Positive predictive values
NPV: Negative positive predictive values
SD: Standard deviation
