# Supplementary material for: Development and validation of a scoring system for predicting cancer patients at risk of extended-spectrum b-lactamase-producing Enterobacteriaceae infections
Source: BMC Infect Dis. 2020 Jul 31;20:558. doi: 10.1186/s12879-020-05280-4 (PMC7393702; doi:10.1186/s12879-020-05280-4)
Supplement: Supplementary file 1 — Additional file 1. [file 12879_2020_5280_MOESM1_ESM.docx]

**Supplementary analysis.**

**Table 1. Supplement.**

**Score distribution in the Validation phase for Blood cultures**

**Score distribution in the Validation phase for Blood cultures**

Score 0 1 2 3 4 5 6 7 Total

non-ESBL-PE 4 23 45 57 14 8 2 1 154

ESBL-PE 0 1 7 9 7 5 1 0 30

Total 4 24 52 66 21 13 3 1 184

Figure 1 Supplement. Receiver Operational Characteristic Curve Analysis with crude score. Blood cultures Validation phase.

Figure 2. Distribution of crude score. Blood cultures Validation phase.
